# High-efficiency derivation of human embryonic stem cell lines using a culture system with minimized trophoblast cell proliferation

**DOI:** 10.1186/s13287-018-0866-5

**Published:** 2018-05-11

**Authors:** Chuti Laowtammathron, Pimjai Chingsuwanrote, Roungsin Choavaratana, Suphadtra Phornwilardsiri, Ketsara Sitthirit, Chidchanok Kaewjunun, Orawan Makemaharn, Papussorn Terbto, Supaporn Waeteekul, Chanchao Lorthongpanich, Yaowalak U-pratya, Pimonwan Srisook, Pakpoom Kheolamai, Surapol Issaragrisil

**Affiliations:** 1 Siriraj Center of Excellence for Stem Cell Research (SiSCR), Faculty of Medicine Siriraj Hospital, Mahidol University, Bangkok, 10700 Thailand; 2 Division of Infertility and Reproductive Biology, Department of Obstetrics and Gynaecology, Faculty of Medicine Siriraj Hospital, Mahidol University, Bangkok, 10700 Thailand; 3 Department of Pathology, Faculty of Medicine Siriraj Hospital, Mahidol University, Bangkok, 10700 Thailand; 4 Division of Medical Genetics, Department of Obstetrics and Gynaecology, Faculty of Medicine Siriraj Hospital, Mahidol University, Bangkok, 10700 Thailand; 5 Division of Hematology, Department of Medicine, Faculty of Medicine Siriraj Hospital, Mahidol University, Bangkok, 10700 Thailand; 6 Division of Cell Biology, Faculty of Medicine, Thammasat University, Pathumthani, 12120 Thailand

**Keywords:** Human embryonic stem cells, Embryo, Clinical grade, Trophoblast cells

## Abstract

**Background:**

Due to their extensive self-renewal and multilineage differentiation capacity, human embryonic stem cells (hESCs) have great potential for studying developmental biology, disease modeling, and developing cell replacement therapy. The first hESC line was generated in 1998 by culturing inner cell mass (ICM) cells isolated from human blastocysts using an immunosurgery technique. Since then, many techniques including mechanical ICM isolation, laser dissection, and whole embryo culture have been used to derive hESC lines. However, the hESC derivation efficiency remains low, usually less than 50%, and it requires a large number of human embryos to derive a significant number of hESC lines. Due to a shortage of and restricted access to human embryos, a novel approach with better hESC derivation efficiency is badly needed to decrease the number of embryos used.

**Methods:**

We hypothesized that the low hESC derivation efficiency might be due to extensive proliferation of trophoblast (TE) cells which could interfere with ICM proliferation. We therefore developed a methodology to minimize TE cell proliferation by culturing ICM in a feeder-free system for 3 days before transferring them onto feeder cells.

**Results:**

This minimized trophoblast cell proliferation (MTP) technique could be successfully used to derive hESCs from normal, abnormal, and frozen–thawed embryos with better derivation efficiency of more than 50% (range 50–100%; median 70%).

**Conclusions:**

We successfully developed a better hESC derivation methodology using the “MTP” culture system. This methodology can be effectively used to derive hESCs from both normal and abnormal embryos under feeder-free conditions with higher efficiency when compared with other methodologies. With this methodology, large-scale production of clinical-grade hESCs is feasible.

**Electronic supplementary material:**

The online version of this article (10.1186/s13287-018-0866-5) contains supplementary material, which is available to authorized users.

## Background

Human embryonic stem cells (hESCs) have the capability to self-renew indefinitely in culture while retaining their ability to differentiate to all cell types. hESCs not only play important roles in basic research in developmental biology, disease pathogenesis, and gene function, but also serve as a valuable model for drug screening and tissue transplantation. At present, more than 300 hESC lines have been eligibly registered to the National Institutes of Health Human Embryonic Stem Cell Registration (http://grants.nih.gov/stem_cells/registry/current.htm). Most hESCs are usually derived from discarded embryos or embryos donated from a couple who have completed in vitro fertilization (IVF) treatments and have no desire to utilize the remaining embryos for transplantation [1, 2]. To generate hESC lines for various research and clinical applications, a large number of human embryos are usually required (Additional file 1: Table S1) [2, 3]. To derive hESCs, immunosurgery, mechanical ICM dissection (MID), and whole embryo culture (WEC) techniques are developed. Immunosurgery is a method for removing TE cells, the outer cell layer of blastocysts, through complement-dependent antibody cytotoxicity [4]. Because an animal-derived antibody is required for immunosurgery, this technique is therefore unsuitable for deriving clinical-grade hESCs. Exposing hESCs to animal products could increase risks of zoonosis and immune reaction against contaminated nonhuman molecules, such as sialic acid *N*-Glycolylneuraminic acid (Neu5Gc), resulting in the rejection of transplanted hESCs [3, 5–12]. Derivation of hESCs with either MID or WEC can be used to derive clinical-grade hESCs. However, the hESC derivation efficiency with those methods is generally low [1, 13–16]. Due to a shortage of and restricted access to human embryos, a more efficient procedure for deriving hESCs under xeno-free conditions is critically needed.

Although induced pluripotent stem cells (iPSCs) generated by epigenetic reprogramming of adult somatic cells can be used as a substitute for hESCs for studying basic biology and pathophysiology of human diseases [17], several recent studies reported that many iPSC lines have acquired various genetic mutations during the course of their generation and expansion that might compromise their clinical use [18–28].

In the present study, we successfully develop better a hESC derivation methodology using a culture system called “minimized trophoblast cell proliferation” (MTP). This methodology can be effectively used to derive hESCs from both normal and abnormal embryos under feeder-free conditions with higher efficiency when compared with other methodologies.

## Methods

### Ethical permission for human embryos used

The study protocol was approved by Siriraj Institutional Review Board, Faculty of Medicine, Siriraj Hospital (SIRB), Mahidol University (Si338/2013). All experiments were performed under the guidelines and regulations of SIRB, Mahidol University. The human embryos used in this study were obtained from the infertility unit, Siriraj Hospital. Informed consent was obtained from all couples that donated spare embryos following in vitro fertilization (IVF) treatment. Before giving consent, the couples were provided with all of the necessary information about the research project and they were made aware of the sensitive nature of the study. Only embryos with genetic abnormalities, diagnosed by performing blastomere biopsy and preimplantation genetic diagnosis (PGD), were used for embryonic stem cell derivation.

### Derivation of human embryonic stem cells

#### Whole embryo culture method

**Fig. 1:**
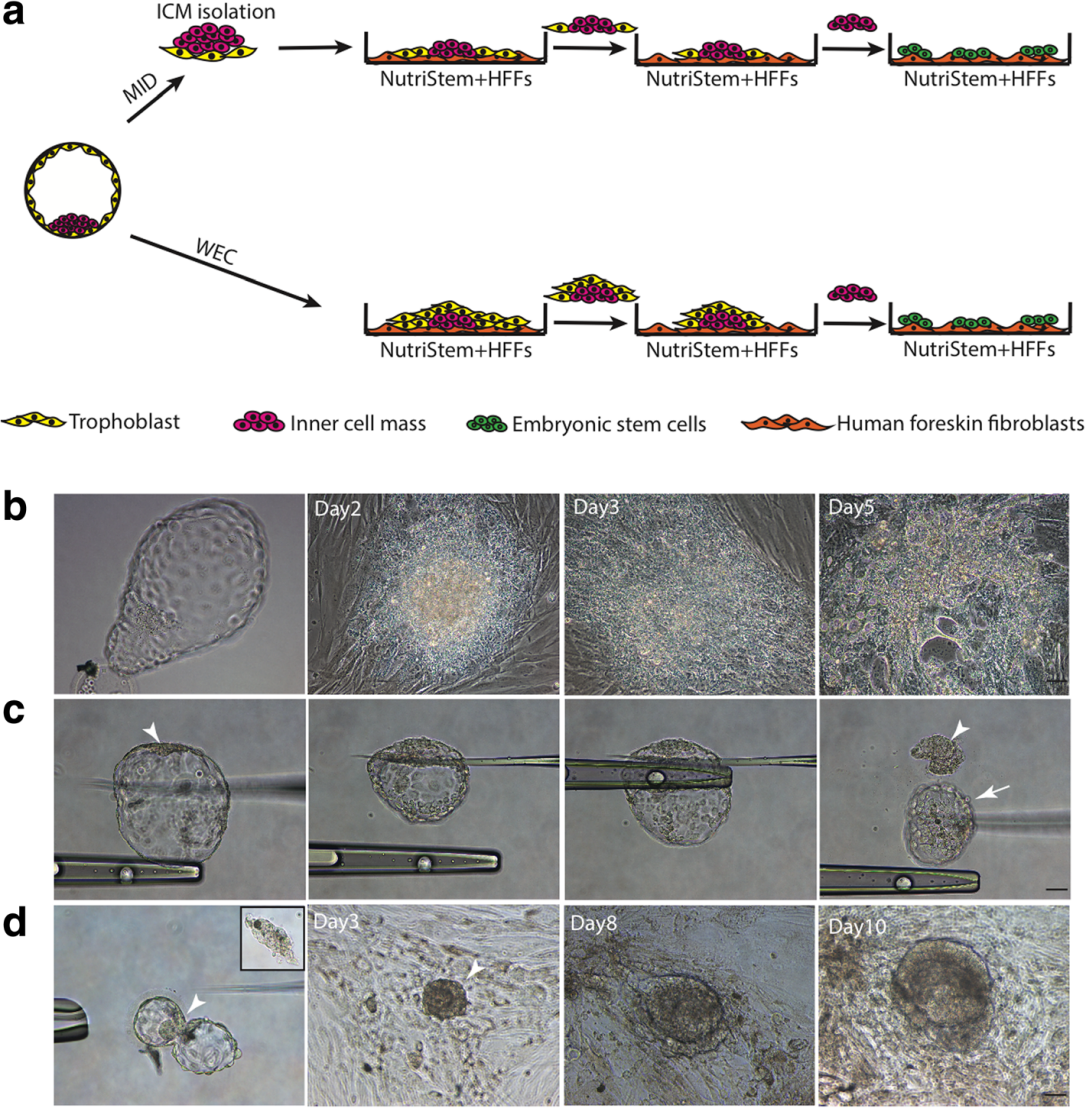
Derivation of hESCs by WEC and MID. **a** Process of hESC derivation from human blastocysts by WEC and MID. **b** Derivation of hESCs by WEC: culture whole embryo on HFFs without ICM isolation. **c, d** ICM (arrowheads) mechanically separated from TE cells (arrow) by glass pipette (**c**) before transferring onto HFFs for further expansion (**d**). Scale bar: 50 μm. MID: mechanical ICM dissection, ICM: inner cell mass, WEC: whole embryo culture, HFFs: human foreskin fibroblasts

The zona pellucida (ZP) was removed by incubating the embryos with 0.1% (w/v) pronase for 5 min followed by washing in NutriStem medium (Stemgent, USA). The ZP-free embryos were then cultured on human foreskin fibroblasts (HFFs) in NutriStem media under hypoxic conditions (a humidified atmosphere of 5%O_2_, 5%CO_2_, and 90%N_2_) (Fig. 1a, b).

#### Mechanical ICM dissection method

The ZP-free embryos were transferred to the micromanipulator for ICM isolation. Firstly, the embryos were suspended in a micro-drop of NutriStem medium and their ICMs were adjusted to the 12 o’clock position. A fine glass needle was then pressed on top of the embryos in the region under their ICMs while the holding pipette was placed underneath the needle and moved back and forth several times to tear off TE cells (Fig. 1c). The isolated ICM was then cultured on HFFs in Nutristem medium under hypoxic conditions (Fig. 1a, d).

#### Minimized trophoblast cell proliferation method

**Fig. 2:**
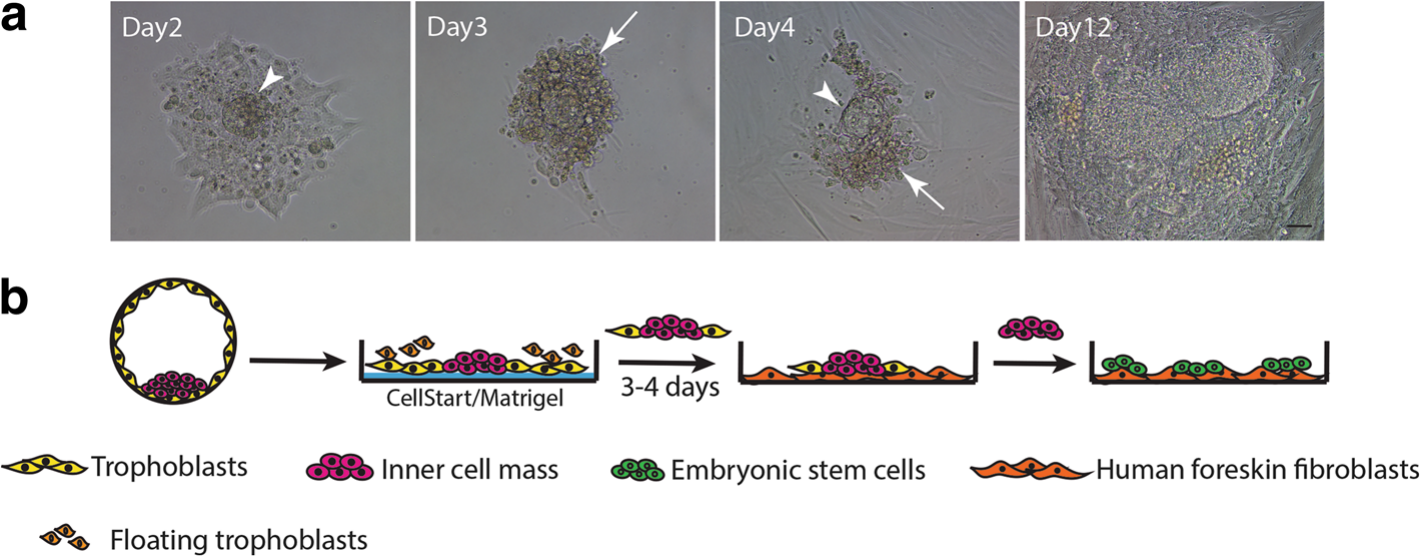
Derivation of hESCs by MTP. **a** On day 2 after ICM isolation, prominent ICM clump (arrowheads) surrounded with proliferating TE cells observed. Under feeder-free conditions using either CELLstart or Matrigel, most proliferating TE cells were degenerated on culture day 3 (arrows) while remaining ICM clump was transferred to fresh HFFs for further expansion. **b** Procedure of hESC derivation by MTP method. Scale bar: 50 μm

The ICMs isolated by MID were cultured on plates coated with either CELLstart (Gibco, USA) or Matrigel (Corning, USA) in Nutristem medium under hypoxic conditions. After 3 days of culture, the ICM clump was mechanically separated from the surrounding TE cells and transferred to a fresh culture plate containing HFFs (Fig. 2b). At this stage, half of the culture medium was replaced by fresh medium every day throughout the entire culture period. The culture was observed daily and the cells were mechanically subcultured when their colonies reached optimal size.

### hESC culture

hESC colonies were cut into small pieces with a fine glass needle and transferred onto freshly prepared gamma-irradiated HFFs. The cells were then cultured in NutriStem medium (Biological Industries, USA) under hypoxic conditions and were subcultured every 4–5 days when their colonies reached optimal size. For the feeder-free system, hESC colonies were cultured on a Matrigel-coated plate in NutriStem media under hypoxic conditions and subcultured every 4–5 days using Versene treatment (Life Technologies, USA).

### Karyotype analysis

hESCs were expanded under feeder-free conditions until their density reached 100% confluence. At this stage, the cells were harvested and sent to Siriraj Central Cytogenetic Laboratory, Faculty of Medicine, Siriraj Hospital, Mahidol University for karyotyping.

### Studying the expression of pluripotent marker genes by RT-PCR

Expression of *OCT4*, *SOX2*, and *NANOG* genes was determined by reverse transcription PCR (RT-PCR). Briefly, total RNA was isolated by Trizol Reagent (Invitrogen, USA), according to the manufacturer’s instructions, and 1 μg of RNA was converted to cDNA by the RevertAid First Strand cDNA Synthesis Kit (Thermo Scientific, USA). The PCR was then performed and the results were analyzed by agarose gel electrophoresis. The primer sequences are presented in Additional file 1: Table S3.

### Immunofluorescence staining

Cells were fixed with 4% (w/v) paraformaldehyde in PBS and their membranes were permeabilized with 0.1% (w/v) Triton X-100 in PBS. At this stage, the cells were incubated with 3% (w/v) BSA in PBS at room temperature for 2 h to prevent nonspecific antibody reaction before incubating with mouse antibodies against human NANOG (1:100; Millipore), OCT4 (1:100; Santa Cruz), SOX2 (1:200; Millipore), SSEA4 (1:100; Millipore), TRA1–60 (1:100; Millipore), and TRA-1-81 (1:100; Millipore) at 4 °C overnight. After incubation with primary antibodies, the cells were washed twice with PBS and further incubated with appropriate secondary antibodies (1:500) at room temperature for 1 h. The nuclei were visualized by staining with Hoechst33342 (Life Technologies, USA) and the cells were studied by fluorescent microscopy.

### In vitro differentiation

hESCs were harvested by incubation with dispase and transferred to low-adherent culture dishes (Corning, USA) containing DMEM (Invitrogen, USA) supplemented with 20% (v/v) serum replacement (Invitrogen, USA), 10 mM non-essential amino acids (Invitrogen, USA), 55 mM β-mercaptoethanol (Invitrogen, USA), 2 mM l-GlutaMAX (Invitrogen, USA), and 50 μg/ml penicillin/streptomycin (Millipore, USA) to form embryoid bodies (EBs). The medium was replaced every 2–3 days. On culture day 7, EBs were transferred to gelatin-coated plates and allowed to spontaneously differentiated for a further 2 weeks. The expression of smooth muscle actin (Abcam, USA), α-fetoprotein (Calbiochem, USA), and NESTIN (Millipore, USA), markers for three primitive germ layers, was determined by immunofluorescence.

### Teratoma formation

Approximately 1 × 10^7^ hESCs were suspended in 30% (v/v) Matrigel and transplanted intramuscularly into hind legs of 6–8-week-old nude mice. At 8–10 weeks post transplant, the fully formed teratomas were harvested and fixed with 4% (w/v) paraformaldehyde in PBS, embedded in paraffin, sectioned, and stained with hematoxylin and eosin for histological analysis.

### Detection of α-thalassemia 1 SEA mutation

Detection of α-thalassemia 1 SEA mutation was performed as described previously by Winichagoon et al. [29]. Briefly, one or two blastomeres were removed from the embryos when they reached the eight-cell stage and transferred to a 0.2-ml reaction tube containing 2.5 μl lysis buffer and 50 μg/ml proteinase K (Invitrogen, USA). PCR was then performed to detect the mutation of α-globin genes. The primer sequences are presented in Additional file 1: Table S3.

## Results

### Derivation of hESCs by whole embryo culture and mechanical ICM dissection

To compare the efficiency of our MTP method with previously available hESC derivation methods, we firstly derived hESCs under feeder-based conditions using two standard hESC derivation methods, WEC and MID (Fig. 1a). For WEC, the zona pellucida (ZP) of three human blastocysts was removed by incubating the embryos with pronase enzyme. The zona-free embryos were then cultured on irradiated human foreskin fibroblasts (HFFs) in Nutristem medium, a commercial GMP-certified, xeno-free medium for hESC culture. After 3 days of culture, flattened outgrowths from the embryos were observed (Fig. 1b). However, most of those outgrowths mainly consisted of TE cells which disrupted the ICM organization. On culture day 5, no ICM outgrowth was observed and no hESC line was derived from those three embryos (Fig. 1b and Table 1).

**Table 1:** Efficiency of hESC derivation using WEC, MID, and MTP techniques^a^

	Number of embryos^b^	Outgrowth (day 3)	Number (%) of established hESC lines
WEC	3	3	0
MID	6	6	2 (33)
MTP	10	10	7 (70)

*hESC* human embryonic stem cell, *WEC* whole embryo culture, *MID* mechanical ICM dissection, *ICM* inner cell mass, *MTP* minimized trophoblast proliferation

^a^Experiments presented were performed by the same personnel

^b^All embryos used were discarded embryos with genetic abnormalities, diagnosed by performing blastomere biopsy and preimplantation genetic diagnosis

For MID, ICM was mechanically isolated from the surrounding TE cells (Fig. 1c). Six ICM clumps were successfully isolated from six blastocysts (three with Hb Bart’s hydrops fetalis, two with trisomy 13, and one with trisomy 18). The isolated ICMs were then cultured on irradiated HFFs in Nutristem medium. After 3 days of culture, several distinct ICM organizations surrounded with a small number of TE cells were observed (Fig. 1d). Only two ICMs with Hb Bart’s hydrops fetalis can be further expanded to generate hESC lines, SiBart1 and SiBart2 (Additional file 2: Figure S1).

### Decrease TE cell proliferation using a feeder-free culture system

It is possible that the feeder cells may promote TE cell proliferation which results in the low hESC derivation efficiency observed with the MID technique. We therefore cultured ICMs on plates coated with CELLstart or Matrigel in Nutristem media under hypoxic conditions without feeder cells. We observed that the ICMs attached to both the CELLstart and Matrigel-coated surfaces within the first day and the ICM clumps were prominent after 2 days of culture. Although TE cell proliferation and expansion was initially observed (Fig. 2a), those TE cells subsequently deteriorated on day 3 or 4. When the ICM clumps were picked and transferred onto fresh HFFs, they further expanded and were ready for passage on culture day 14 while the TE cells were no longer observed. This result indicates that culturing ICMs on CELLstart or Matrigel instead of HFFs can prevent TE cell proliferation and enhance ICM expansion (Fig. 2b).

### Enhancement of hESC derivation using the MTP technique

**Fig. 3:**
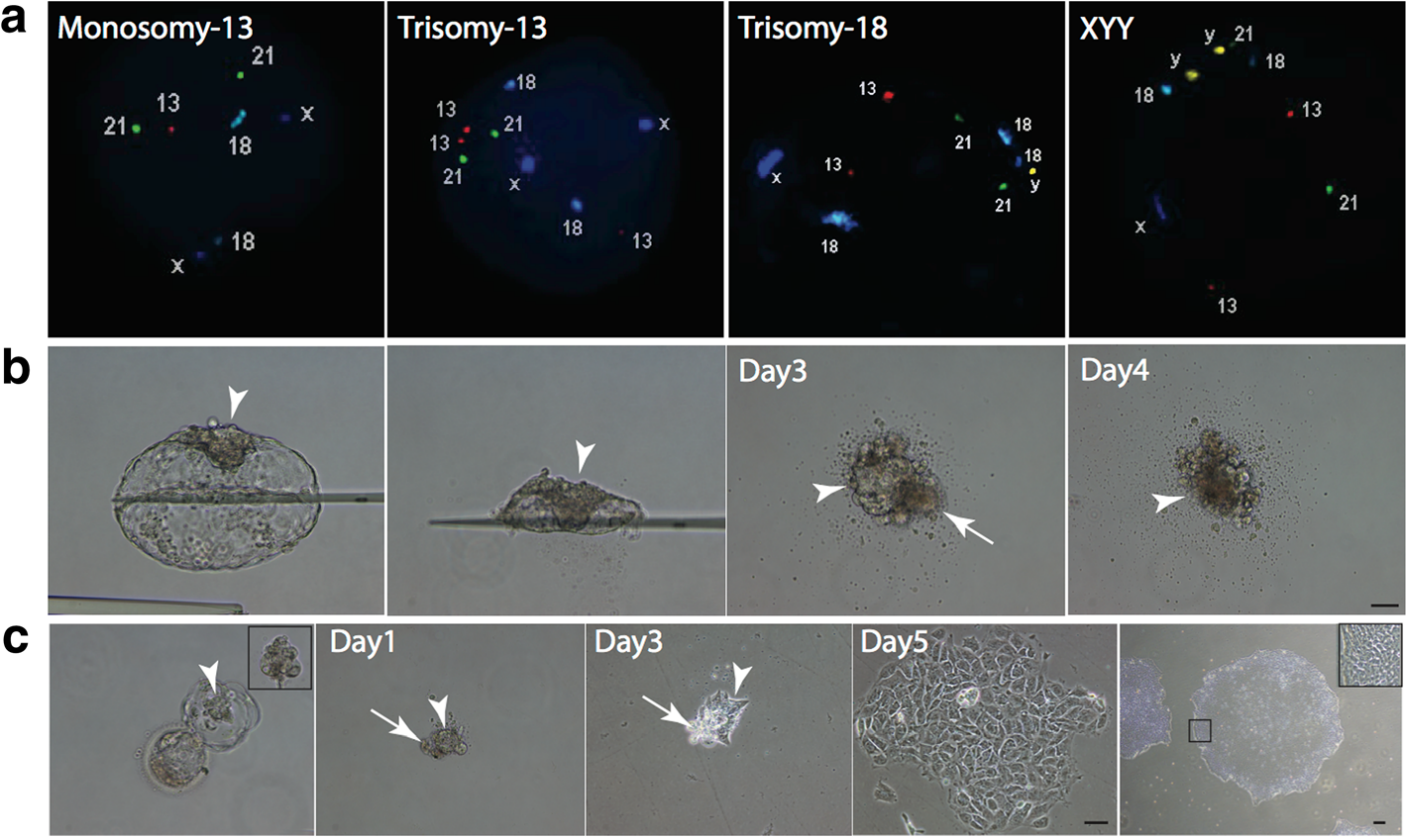
Derivation of hESCs from aneuploid embryos using MTP under a feeder-free system. **a** Eight-cell-stage human embryos subjected to blastomere biopsy and their chromosomal abnormalities determined by fluorescence in-situ hybridization (FISH). **b** Mechanically isolated ICM (arrowheads) with large number of associated TE cells (arrows) detached from Matrigel-coated surface and degenerated after 4 days of culture. **c** Mechanically isolated ICM (arrowheads) carefully stripped of TE cells (arrows) remains attached to Matrigel-coated surface and continuously proliferated to form large hESC colonies while remaining TE cells degenerated. Scale bar: 50 μm

Seven embryos (one parthenogenetic embryo and six aneuploid embryos including two with monosomy 13, two with trisomy 13, one with trisomy 18, and one with XYY syndrome; Fig. 3a) were studied. The ICMs from each embryo were isolated by MID and cultured on Matrigel-coated plates in Nutristem media under hypoxic conditions. On culture day 3, the ICM clumps were separated from the surrounding TE cells and transferred onto HFFs. At this stage, the ICM cells proliferated extensively and five hESC lines were successfully established with a derivation efficiency of 71.4%. Of these five hESC lines, two were derived from monosomy 13 embryos (Si1 and Si2) and one each from XYY embryo (Si3), trisomy 13 embryo (Si4), and parthenogenetic embryo (Si5).

We also used the MTP method to derive hESCs from frozen–thawed embryos. ICMs were isolated from two frozen embryos with 3 pronuclei (3PN) by MID and cultured on Matrigel-coated plates in Nutristem media under hypoxic conditions. One hESC line (Si3PN) was then successfully established with a derivation efficiency of 50% (Additional file 3: Figure S2A). Karyotyping showed that Si3PN exhibited the 69, XXY karyotype (Additional file 3: Figure S2C). We also successfully used the MTP method to derive hESCs from an embryo with Hb Bart’s hydrops fetalis (SiAtha1) (Additional file 3: Figure S2B). Karyotyping reveals that SiAtha1 exhibited the normal 46, XY karyotype (Additional file 3: Figure S2D).

Taken together, the results show that our MTP method enhanced the hESC derivation efficiency from both normal and abnormal embryos. Using the MTP technique, seven hESC lines were successfully derived from 10 embryos with an overall efficiency of 70% (Table 2), the highest among other previous reports (Additional file 1: Table S1), and approximately 3-fold higher than the conventional methods, MID and WEC (Table 1, Additional file 1: Table S2). All hESC lines established in this study expressed typical pluripotent marker genes and proteins, and could differentiate to generate derivatives of all three embryonic germ layers both in vitro and in vivo (Additional file 4: Figure S3).

**Table 2:** hESC lines successfully derived by MTP technique

Type of embryo	Number of embryos	Number (%) of established hESC lines	Name of hESC line
Monosomy 13	2	2 (100)	Si1, Si2
Trisomy 13	2	1 (50)	Si4
47, XYY	1	1 (100)	Si3
Parthenogenetic	1	1 (100)	Si5
3PN (vitrified–thawed)	2	1 (50)	Si3PN
Alpha thalassemia	1	1 (100)	SiAtha1

*hESC* human embryonic stem cell, *MTP* minimized trophoblast proliferation

### Derivation of hESCs in a feeder-free culture system using minimized TE cell contamination

To explore use of the MTP method for deriving hESCs under feeder-free conditions, we cultured the ICMs isolated by MID on Matrigel-coated plates throughout the entire culture period without transferring the cells onto HFFs. TE cells deteriorated after 3 days of culture while the ICM remained intact. However, when the culture was continued under this condition, the ICMs began to degenerate and detached from the culture surface on culture day 5 (Fig. 3b). When the detached ICMs were transferred onto HFFs for further expansion, they did not proliferate, resulting in the termination of culture.

**Fig. 4:**
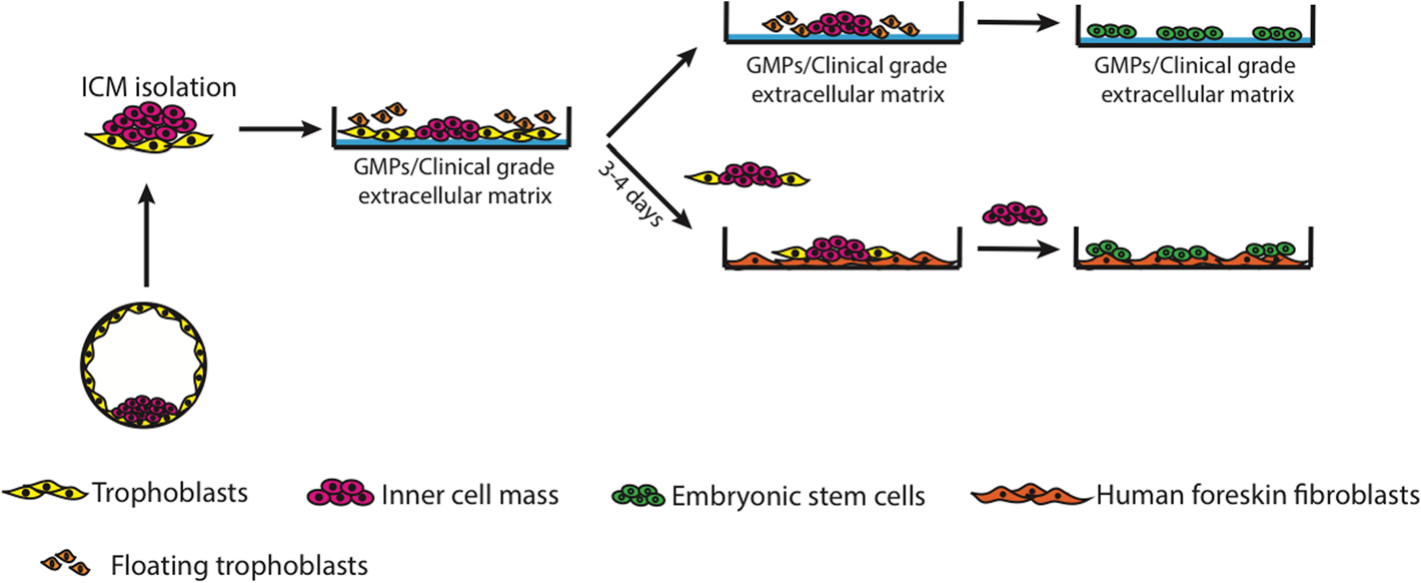
Derivation of clinical-grade hESCs by MTP under feeder-based and feeder-free conditions. To generate clinical-grade hESCs, mechanically isolated ICM initially cultured on GMP/clinical-grade extracellular matrix, such as rLaminin-521 or CELLstart, for a few days before being transferred onto GMP-grade HFFs (for feeder-based system) or fresh matrix-coated plates (for feeder-free system) to generate hESC colonies. ICM: inner cell mass, GMP: good manufacturing practice

Based on this observation, it is possible that an extensive deterioration of TE cells prior to the degeneration of ICMs might release some toxic molecules that exerted deleterious effects on ICM cells. To solve this problem, we tried to minimize the number of contaminated TE cells at the beginning of culture by carefully separating ICMs from the surrounding TE cells in the blastocysts using a fine pulled-glass needle. When those carefully isolated ICMs were cultured on Matrigel-coated plates, they attached to the culture surface and began to proliferate. Using this technique, the numbers of contaminated TE cells in culture were dramatically decreased and the ICM outgrowths proceed even after the remaining TE cells degenerated. After 8 days of culture, the ICM-derived colonies were mechanically dissociated and replated into fresh Matrigel-coated plates. The ICM-derived colonies which exhibited typical hESC morphology (Fig. 3c) could be expanded further and the hESC lines were successfully established. The results demonstrate that an optimized MTP method could be used to derive clinical-grade hESC lines under feeder-free and xeno-free medium (Fig. 4).

## Discussion

Previous reports using either the MID or WEC technique to derive hESCs indicated that TE cell outgrowth could suppress ICM expansion and cause them to degenerate, resulting in the low hESC derivation efficiency [30–32]. In the present study, we successfully developed a more effective hESC derivation technique by minimizing the amount of contaminated TE cells during mechanical ICM dissection and using a feeder-free culture system to limit TE cell proliferation (MTP technique). The idea of using the MTP technique is to reduce TE cell proliferation and allow the ICM to expand during the first few days of culture. At this time, the ICM cells begin to acquire the embryonic stem cell phenotype as demonstrated by the upregulation of genes associated with self-renewal, such as Lin28a [33, 34]. It is of importance that the separation of ICM outgrowth from degenerated TE cells is critical for the successful derivation of hESCs under feeder-free conditions. Allowing degenerated TE cells to contact with ICM outgrowth results in ICM degeneration, possibly due to toxic substances or apoptosis signals released from the degenerating TE cells.

The hESC lines derived from various types of embryos using the MTP technique exhibited typical characteristics and functional properties of hESCs, and some lines (Si3PN and SiAtha1) preserved the original genetic mutations of the embryos that were used to derive them. The MTP technique can also be used to derive hESCs under xeno-free conditions, which is suitable for clinical-grade hESC derivation. We also showed that CELLstart, which is a nonanimal GMP-grade extracellular matrix, can be used to derive hESCs by our MTP technique.

In this study, we used human embryos with various chromosomal abnormalities to derive hESCs. Blastomere biopsy was performed when the embryos reached the eight-cell stage for PGD. Those embryos therefore consisted of approximately 12.5–25% fewer cells than intact embryos [35]. It is controversial whether blastomere biopsy affects embryonic development and the implantation rate. Previous studies demonstrated that blastomere biopsy did not affect blastocyst formation, embryonic implantation, and subsequent development of postimplantation embryos [30–32, 36–41]. However, there were reports suggesting that biopsied embryos showed delayed compaction, generated fewer hatched blastocysts, and had a lower implantation rate (39%) when compared with nonbiopsied embryos [42–47]. Our results showed that blastomere biopsy did not affect the hESC derivation efficiency, indicating that the remaining number of blastomeres after biopsy is sufficient for generating ICM for subsequent hESC derivation by the MTP method.

Surprisingly, all hESC lines derived from four embryos with various chromosomal abnormalities exhibited a normal diploid karyotype (Additional file 5: Figure S4). However, this was not unprecedented as there was a report of deriving normal diploid hESCs from aneuploid embryos [48]. This may be due to a phenomenon called “mosaicism” found in small percentages of eight-cell-stage embryos at the time of PGD [49]. The meiotic nondisjunction occurring during germ cell formation and the mitotic nondisjunction occurring during the early cleavage stage of the embryos are mostly responsible for the mosaicism, resulting in affected embryos with both normal diploid cells and aneuploid cells [50–52] (Additional file 6: Figure S5). It is therefore possible that our four abnormal embryos were affected by mosaicism and the normal diploid cells presented in those embryos might outcompete their aneuploid counterparts during the hESC derivation process, resulting in the generation of hESC lines with a normal karyotype (Si1, Si2, Si3, and Si4).

## Conclusion

We report use of the MTP technique to derive hESCs from normal, disease, frozen, and parthenogenetic embryos with efficiency greater than that described previously. With this MTP technique, hESC lines can be successfully derived under feeder-free conditions in well-defined, xeno-free medium (Fig. 4). This methodology can be used to derive clinical-grade hESCs from a limited number of embryos for future therapeutic applications.

## Additional files

Additional file 1: Supplement information including supplementary Tables S1–S3. (DOCX 39 kb)

Additional file 2: **Figure S1.** Detection of α-thalassemia SEA mutation in SiBart1 and SiBart2 hESCs. For α^0^-thalassemia trait (Trait), PCR products of both normal (194 bp) and mutated α-globin gene (570 bp) detected. SiBart1 (Brt1) and SiBart2 (Brt2) hESCs derived from α^0^-thalassemia embryos only have mutated version of α-globin gene while hESCs derived from normal embryo (Chula2) only have normal version of the gene. (JPG 954 kb)

Additional file 3: **Figure S2.** Derivation of hESCs from diseased and frozen–thawed embryos using MTP. (**A**) ICM clump isolated from a frozen–thawed aneuploid 3PN embryo and cultured on Matrigel-coated plate. After culture for 3 days, most TE cells degenerated (arrowhead) while remaining ICM cells were transferred onto fresh HFFs and expanded further to generate hESCs (Si3PN). (**B**) ICM clump isolated from Hb Bart’s hydrops fetalis embryos and cultured on Matrigel-coated plate. Degenerating TE cells (arrowhead) observed while remaining ICM cells were transferred onto fresh HFFs and expanded further to generate hESCs (SiAtha1). (**C**) Karyotyping results show Si3PN exhibited a triploid (69, XXY) karyotype while (**D**) SiAtha1 exhibited a normal diploid karyotype (46, XY). Scale bar: 50 μm. (JPG 1215 kb)

Additional file 4: **Figure S3.** Characterization of hESC lines derived from MTP. (**A**) Total transcripts *OCT4*, *SOX2*, and *NANOG* of each hESC line extracted and amplified by PCR using specific primer. PCR product of each transcript from different hESC lines subjected to same agarose gel and same exposure. RT-PCR analysis shows that hESC lines derived from MTP expressed core pluripotent genes, *OCT4*, *NANOG*, and *SOX2*. (**B**) Immunofluorescent staining shows hESCs derived from MTP expressed typical hESC marker proteins. (**C**) Spontaneous differentiation of seven hESC cell line-generated derivatives of all three primitive germ layers demonstrated by expression of NESTIN, SMA, and AFP. (**D**) Hematoxylin and eosin staining of teratoma generated from Si1–Si4 and SiAtra1 showed tissues from three primitive germ layers including endoderm, mesoderm, and ectoderm. Scale bar: 50 μm. (ZIP 7514 kb)

Additional file 5: **Figure S4.** Karyotyping analysis of Si1, Si2, Si3, Si4, and Si5 cell lines exhibited a normal diploid karyotype. (JPG 720 kb)

Additional file 6: **Figure S5.** Schematic diagram explaining origin of aneuploid chromosome caused by meiotic and mitotic nondisjunction. For meiotic nondisjunction, chromosomal missegregation occurs during meiosis resulting in aneuploid embryos. For mitotic nondisjunction, chromosome missegregation occurs during mitotic cell divisions resulting in mosaicism of normal diploid and aneuploid cells in embryo. For Si3PN, aneuploid chromosome was originated from meiotic nondisjunction, while aneuploid chromosomes of Si1–Si4 likely occurred from mitotic no-disjunction during second or third cell division of the embryos. (JPG 1491 kb)

## Abbreviations

cDNA: Complementary DNA
GMP: Good manufacturing practice
Hb: Hemoglobin
hESC: Human embryonic stem cell
HFF: Human foreskin fibroblast
ICM: Inner cell mass
iPSC: Induced pluripotent stem cell
IVF: In vitro fertilization
MID: Mechanical ICM dissection
MTP: Minimized trophoblast cell proliferation
Neu5Gc: *N*-Glycolylneuraminic acid
PBS: Phosphate buffer saline
PGD: Preimplantation genetic diagnosis
RT-PCR: Reverse transcription polymerase chain reaction
SEA: Southeast Asia
SIRB: Siriraj Institutional Review Board, Faculty of Medicine, Siriraj Hospital
TE: Trophoblast
WEC: Whole embryo culture
ZP: Zona pellucida
