# Supplementary material for: High-efficiency derivation of human embryonic stem cell lines using a culture system with minimized trophoblast cell proliferation
Source: Stem Cell Res Ther. 2018 May 11;9:138. doi: 10.1186/s13287-018-0866-5 (PMC5948903; doi:10.1186/s13287-018-0866-5)
Supplement: Supplementary file 1 — Supplement information including supplementary Tables S1–S3. (DOCX 39 kb) [file 13287_2018_866_MOESM1_ESM.docx]

**High efficiency derivation of human embryonic stem cell lines using culture system with minimizing trophoblast cell proliferation**

Chuti Laowtammathron^1^, Pimjai Chingsuwanrote^1^, Roungsin Choavaratana^2^, Suphadtra Phornwilardsiri^2^, Ketsara Sitthirit^2^, Chidchanok Kaewjunun^2^, Orawan Makemaharn^2^, Papussorn Terbto^3^, Supaporn Waeteekul^4^,Chanchao Lorthongpanich^1^, Yaowalak U-pratya^1,5^, Pimonwan Srisook^1^, Pakpoom Kheolamai^1,6^, Surapol Issaragrisil^1,5,^*

*Correspondence: [surapolsi@gmail.com](mailto:surapolsi@gmail.com) ^1^Siriraj Center of Excellence for Stem Cell Research (SiSCR), Faculty of Medicine Siriraj Hospital, Mahidol University, Bangkok, 10700, Thailand. ^2^Division of Infertility and Reproductive Biology, Department of Obstetrics and Gynaecology, Faculty of Medicine Siriraj Hospital, Mahidol University, Bangkok, 10700, Thailand.  ^3^Department of Pathology, Faculty of Medicine Siriraj Hospital, Mahidol University, Bangkok, 10700, Thailand. ^4^Division of Medical Genetics, Department of Obstetrics & Gynaecology, Faculty of Medicine Siriraj Hospital, Mahidol University, Bangkok, 10700, Thailand.  ^5^Division of Hematology, Department of Medicine, Faculty of Medicine Siriraj Hospital, Mahidol University, Bangkok, 10700, Thailand., ^6^Division of Cell Biology, Faculty of Medicine, Thammasat University, Pathumthani, 12120, Thailand.

**Supplementary Table**

**Table S1**. Comparison of the hESC derivation efficiency of various methods.

| **Method** | **Number of blastocyst** | **Number of cell line** | **hESC derivation efficiency** | **Authors** |
| --- | --- | --- | --- | --- |
| **Immunosurgery** | 14 | 5 | 35% | Thomson et al., 1998 [1] |
|  | 97 | 17 | 18% | Cowan et al., 2004 [2] |
|  | 7 | 1 | 14% | Stojkovic et al., 2004 [3] |
|  | 5 | 1 | 20% | Klimanskaya et al., 2005 [4]  (FF) |
|  | 8 | 4 | 50% | Lai et al., 2015 [5] |
| **MID** | 19 | 4 | 21% | Crook et al., 2007 [6] |
|  | 236 | 30 | 8% | Strom et al., 2010 [7] |
| **Laser-assisted ICM dissection** | 19 | 11 | 58% | Chen et al., 2009 [8] |
|  | 23 | 7 | 3% | Tannenbaum et al., 2012 [9] |
| **WEC** | 17 | 4 | 23% | Crook et al., 2007 [6] |
|  | 94 | 8 | 8.5% | Lerou et al., 2008 [10] |
|  | 11 | 1 | 9% | Eremeev et al., 2009 [11]  (FF) |
| **MTP** | 10 | 7 | 70% | Laowtammathron et al.  (Current study) |

WEC: Whole embryo culture; MID: Mechanical ICM dissection;
MTP: Minimized trophoblast cell proliferation; FF: Feeder-free

**Table S2**. hESC derivation efficiency of MTP method under feeder-based and feeder-free system.

|  | **No.Embryo** | **Outgrowth** | **Stable hESC line (%)** |
| --- | --- | --- | --- |
| Feeder-based system | 9 | 9 | 2 (22.2) |
| Feeder-free system | 10 | 10 | 7 (70) |

**Table S3**. List of PCR primers used in this study.

|  |  | **Forward primer** | **Reverse primer** |
| --- | --- | --- | --- |
| Pluripotent  genes | *OCT-4*: | GTTGATCCTCGGACCTGGCTA | GGTTGCCTCTCACTCGGTTCT |
|  | *NANOG*: | GTCTTCTGCTGAGATGCCTCACA | CTTCTGCGTCACACCATTGCTAT |
|  | *SOX2*: | GGGAAATGGGAGGGGTGCAAAAGAGG | TTGCGTGAGTGTGGATGGGATTGGTG |
| α-globin gene with SEA mutation | A4: | GGGGCGCCTTGGGGAGGTTC | A1B: GTTCCCTGAGCCCCGACACG |
|  |  |  | A9: ATATATGGGTCTGGAAGTGTATC |
|  |  |  |  |

**References**

1. Thomson JA, Itskovitz-Eldor J, Shapiro SS, Waknitz MA, Swiergiel JJ, Marshall VS, et al. Embryonic stem cell lines derived from human blastocysts. Science (New York, NY). 1998;282(5391):1145-7.

2. Cowan CA, Klimanskaya I, McMahon J, Atienza J, Witmyer J, Zucker JP, et al. Derivation of embryonic stem-cell lines from human blastocysts. The New England journal of medicine. 2004;350(13):1353-6.

3. Stojkovic M, Lako M, Stojkovic P, Stewart R, Przyborski S, Armstrong L, et al. Derivation of human embryonic stem cells from day-8 blastocysts recovered after three-step in vitro culture. Stem cells. 2004;22(5):790-7.

4. Klimanskaya I, Chung Y, Meisner L, Johnson J, West MD, Lanza R. Human embryonic stem cells derived without feeder cells. Lancet (London, England). 2005;365(9471):1636-41.

5. Lai D, Wang Y, Sun J, Chen Y, Li T, Wu Y, et al. Derivation and characterization of human embryonic stem cells on human amnion epithelial cells. Scientific reports. 2015;5:10014.

6. Crook JM, Peura TT, Kravets L, Bosman AG, Buzzard JJ, Horne R, et al. The generation of six clinical-grade human embryonic stem cell lines. Cell stem cell. 2007;1(5):490-4.

7. Strom S, Holm F, Bergstrom R, Stromberg AM, Hovatta O. Derivation of 30 human embryonic stem cell lines--improving the quality. In vitro cellular & developmental biology Animal. 2010;46(3-4):337-44.

8. Chen AE, Egli D, Niakan K, Deng J, Akutsu H, Yamaki M, et al. Optimal timing of inner cell mass isolation increases the efficiency of human embryonic stem cell derivation and allows generation of sibling cell lines. Cell stem cell. 2009;4(2):103-6.

9. Tannenbaum SE, Turetsky TT, Singer O, Aizenman E, Kirshberg S, Ilouz N, et al. Derivation of xeno-free and GMP-grade human embryonic stem cells--platforms for future clinical applications. PloS one. 2012;7(6):e35325.

10. Lerou PH, Yabuuchi A, Huo H, Takeuchi A, Shea J, Cimini T, et al. Human embryonic stem cell derivation from poor-quality embryos. Nature biotechnology. 2008;26(2):212-4.

11. Eremeev AV, Svetlakov AV, Polstianoy AM, Bogomazova AN, Philonenko ES, Sheina YI, et al. Derivation of a novel human embryonic stem cell line under serum-free and feeder-free conditions. Doklady biological sciences : proceedings of the Academy of Sciences of the USSR, Biological sciences sections / translated from Russian. 2009;426:293-5.
