# Supplementary figures and images for: High-efficiency derivation of human embryonic stem cell lines using a culture system with minimized trophoblast cell proliferation
Source: Stem Cell Res Ther. 2018 May 11;9:138. doi: 10.1186/s13287-018-0866-5 (PMC5948903; doi:10.1186/s13287-018-0866-5)

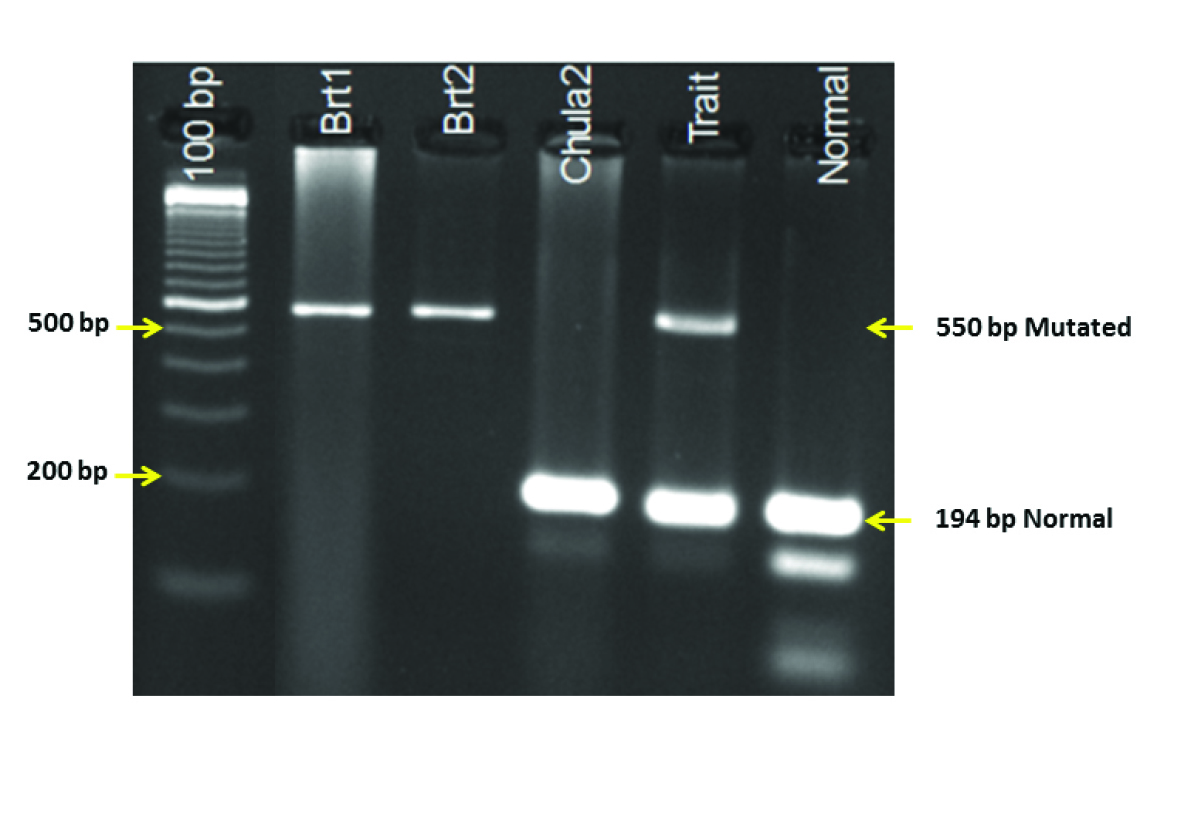

Supplement: Supplementary file 2 — Figure S1. Detection of α-thalassemia SEA mutation in SiBart1 and SiBart2 hESCs. For α0-thalassemia trait (Trait), PCR products of both normal (194 bp) and mutated α-globin gene (570 bp) detected. SiBart1 (Brt1) and SiBart2 (Brt2) hESCs derived from α0-thalassemia embryos only have mutated version of α-globin gene while hESCs derived from normal embryo (Chula2) only have normal version of the gene. (JPG 954 kb) [file 13287_2018_866_MOESM2_ESM.jpg]

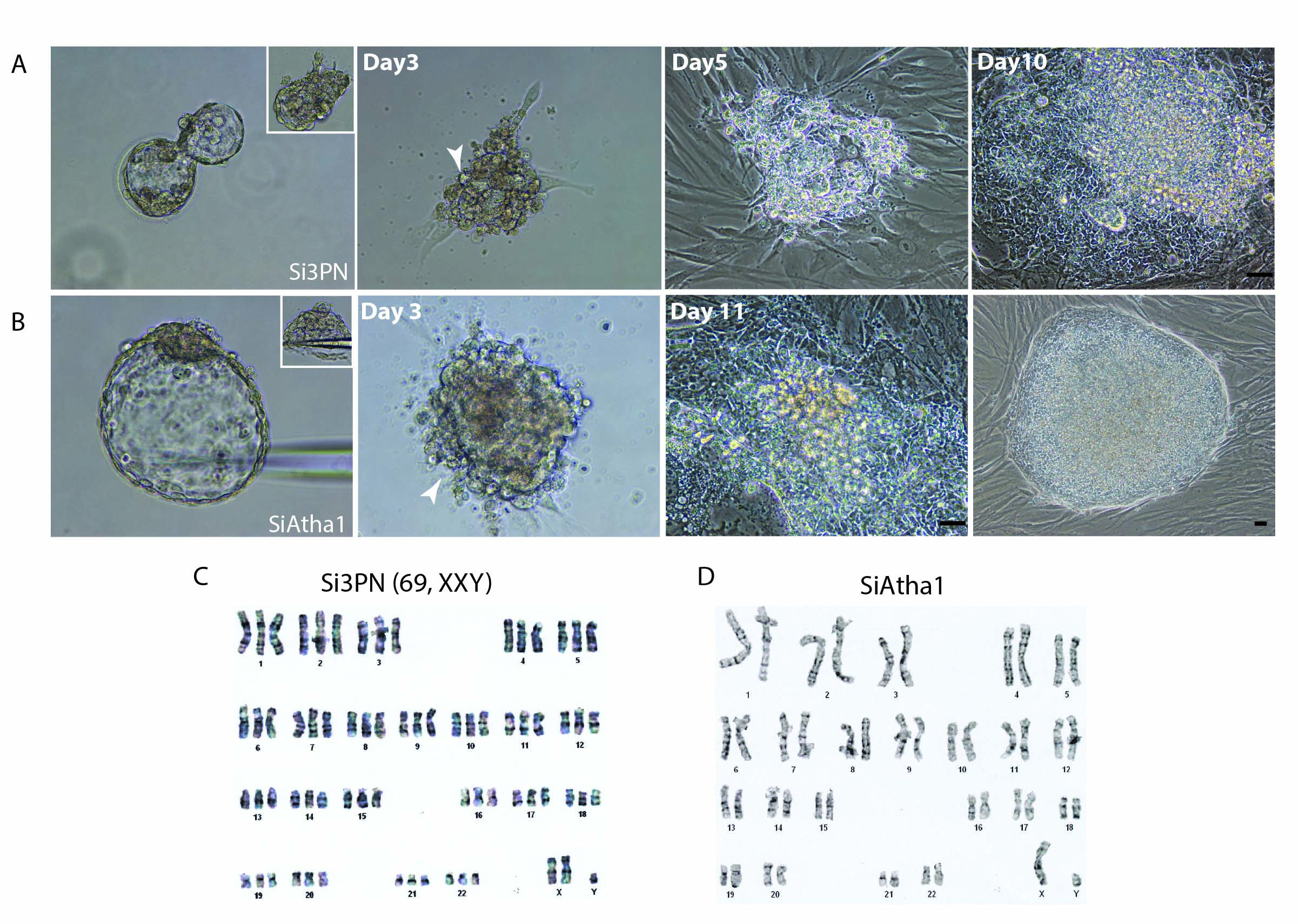

Supplement: Supplementary file 3 — Figure S2. Derivation of hESCs from diseased and frozen–thawed embryos using MTP. (A) ICM clump isolated from a frozen–thawed aneuploid 3PN embryo and cultured on Matrigel-coated plate. After culture for 3 days, most TE cells degenerated (arrowhead) while remaining ICM cells were transferred onto fresh HFFs and expanded further to generate hESCs (Si3PN). (B) ICM clump isolated from Hb Bart’s hydrops fetalis embryos and cultured on Matrigel-coated plate. Degenerating TE cells (arrowhead) observed while remaining ICM cells were transferred onto fresh HFFs and expanded further to generate hESCs (SiAtha1). (C) Karyotyping results show Si3PN exhibited a triploid (69, XXY) karyotype while (D) SiAtha1 exhibited a normal diploid karyotype (46, XY). Scale bar: 50 μm. (JPG 1215 kb) [file 13287_2018_866_MOESM3_ESM.jpg]

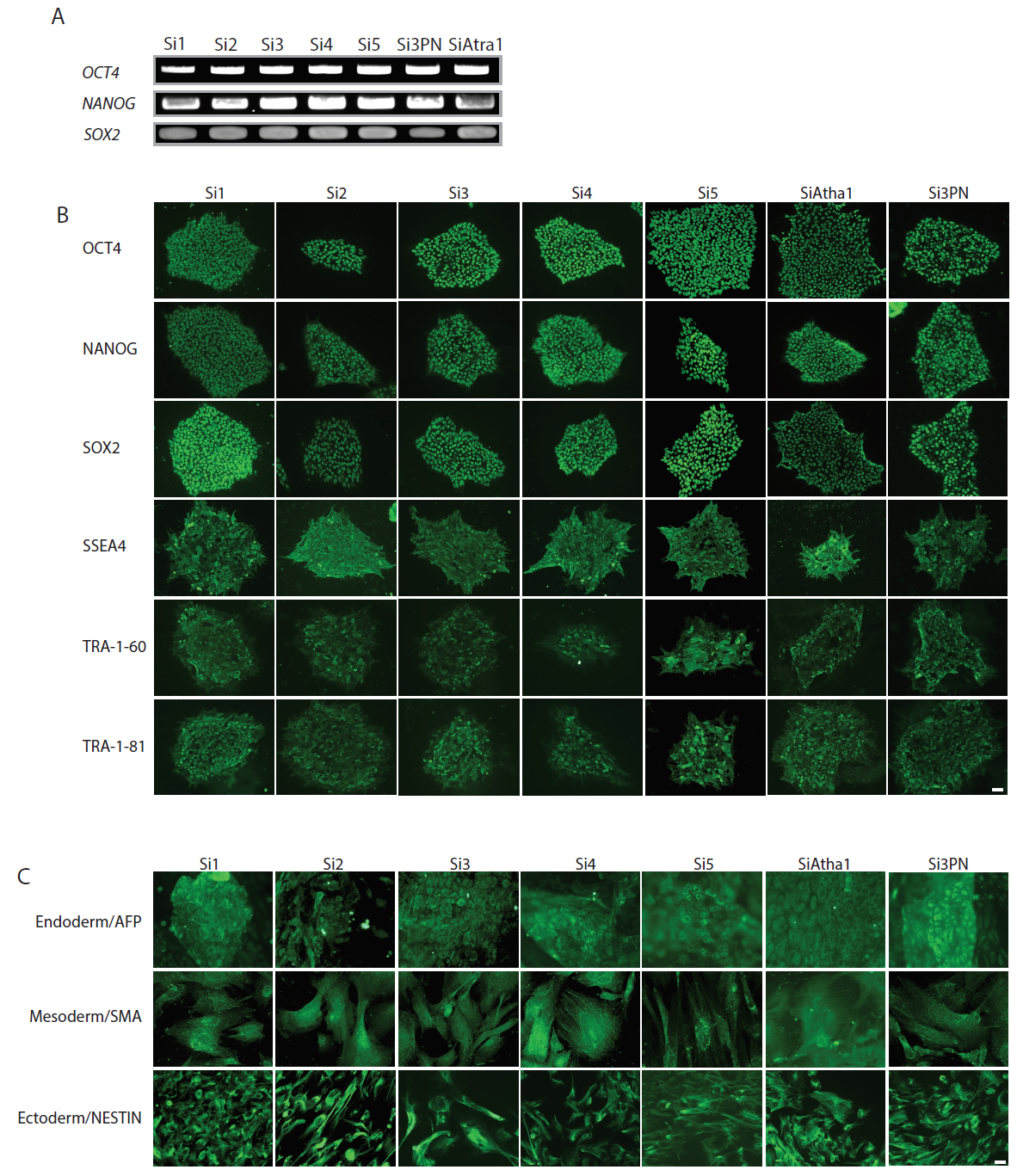

Supplement: Supplementary file 4 — Figure S3. Characterization of hESC lines derived from MTP. (A) Total transcripts OCT4, SOX2, and NANOG of each hESC line extracted and amplified by PCR using specific primer. PCR product of each transcript from different hESC lines subjected to same agarose gel and same exposure. RT-PCR analysis shows that hESC lines derived from MTP expressed core pluripotent genes, OCT4, NANOG, and SOX2. (B) Immunofluorescent staining shows hESCs derived from MTP expressed typical hESC marker proteins. (C) Spontaneous differentiation of seven hESC cell line-generated derivatives of all three primitive germ layers demonstrated by expression of NESTIN, SMA, and AFP. (D) Hematoxylin and eosin staining of teratoma generated from Si1–Si4 and SiAtra1 showed tissues from three primitive germ layers including endoderm, mesoderm, and ectoderm. Scale bar: 50 μm. (ZIP 7514 kb) [file 13287_2018_866_MOESM4_ESM.zip › Supplement S3.jpg]

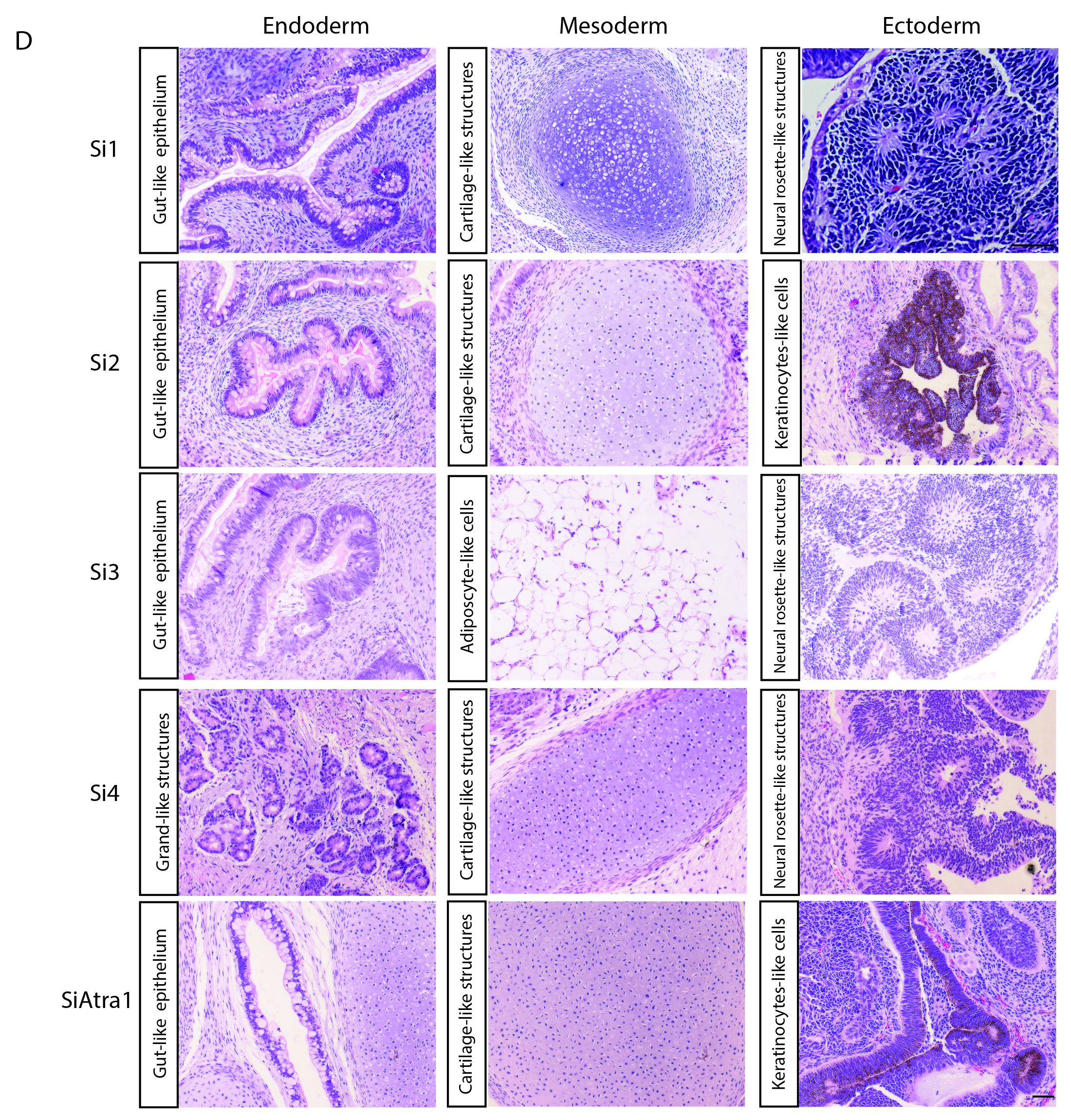

Supplement: Supplementary file 4 — Figure S3. Characterization of hESC lines derived from MTP. (A) Total transcripts OCT4, SOX2, and NANOG of each hESC line extracted and amplified by PCR using specific primer. PCR product of each transcript from different hESC lines subjected to same agarose gel and same exposure. RT-PCR analysis shows that hESC lines derived from MTP expressed core pluripotent genes, OCT4, NANOG, and SOX2. (B) Immunofluorescent staining shows hESCs derived from MTP expressed typical hESC marker proteins. (C) Spontaneous differentiation of seven hESC cell line-generated derivatives of all three primitive germ layers demonstrated by expression of NESTIN, SMA, and AFP. (D) Hematoxylin and eosin staining of teratoma generated from Si1–Si4 and SiAtra1 showed tissues from three primitive germ layers including endoderm, mesoderm, and ectoderm. Scale bar: 50 μm. (ZIP 7514 kb) [file 13287_2018_866_MOESM4_ESM.zip › Supplement S3 Cont..jpg]

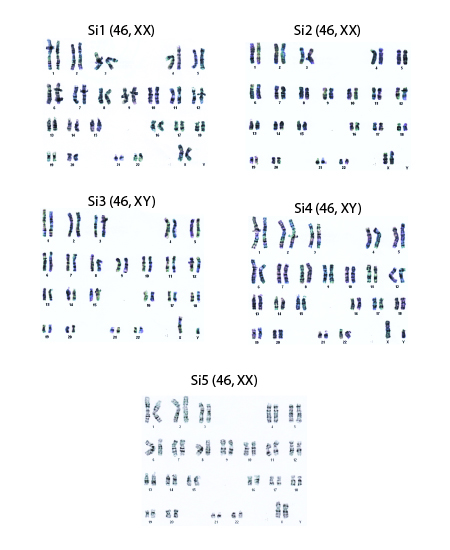

Supplement: Supplementary file 5 — Figure S4. Karyotyping analysis of Si1, Si2, Si3, Si4, and Si5 cell lines exhibited a normal diploid karyotype. (JPG 720 kb) [file 13287_2018_866_MOESM5_ESM.jpg]

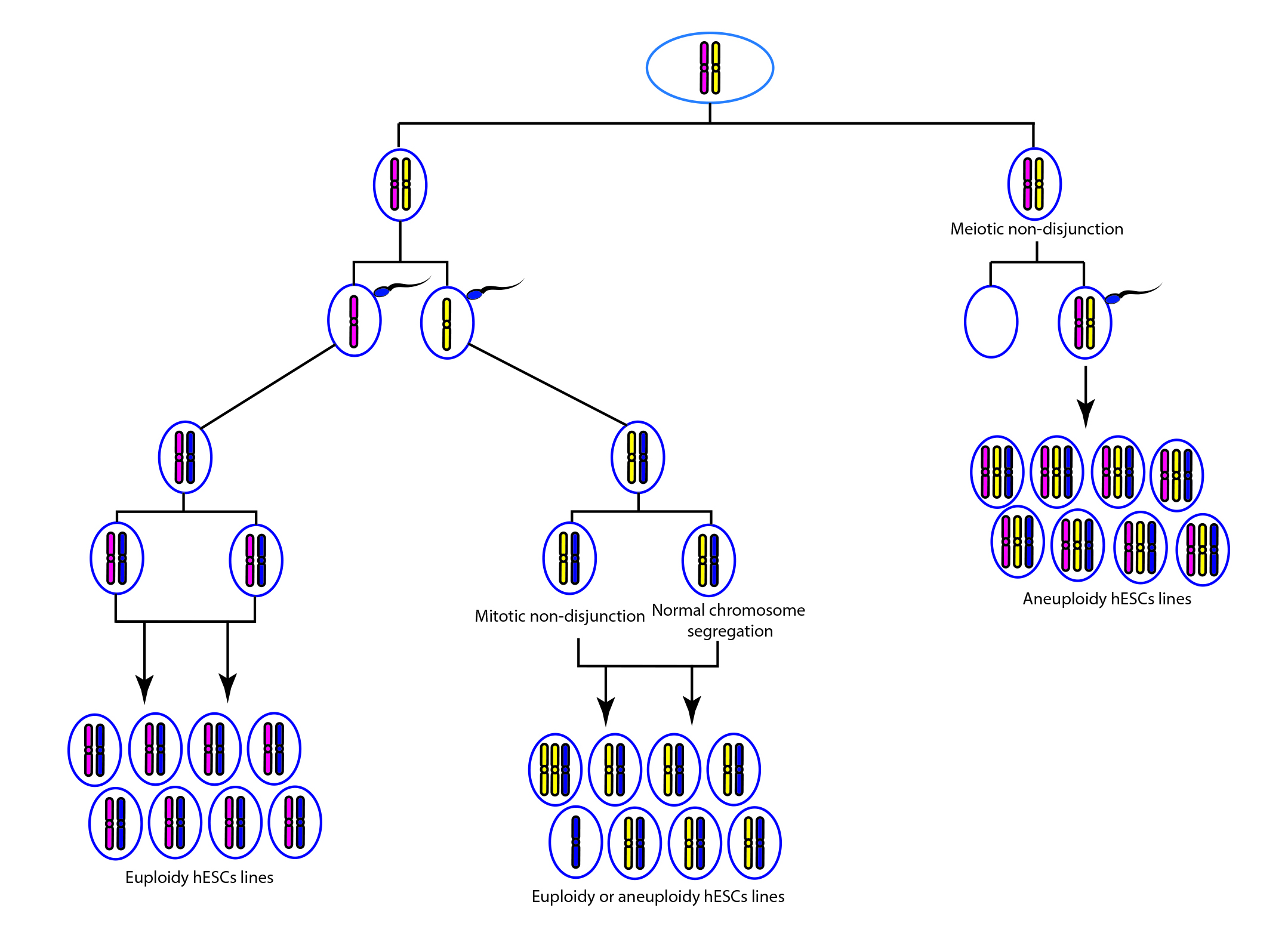

Supplement: Supplementary file 6 — Figure S5. Schematic diagram explaining origin of aneuploid chromosome caused by meiotic and mitotic nondisjunction. For meiotic nondisjunction, chromosomal missegregation occurs during meiosis resulting in aneuploid embryos. For mitotic nondisjunction, chromosome missegregation occurs during mitotic cell divisions resulting in mosaicism of normal diploid and aneuploid cells in embryo. For Si3PN, aneuploid chromosome was originated from meiotic nondisjunction, while aneuploid chromosomes of Si1–Si4 likely occurred from mitotic no-disjunction during second or third cell division of the embryos. (JPG 1491 kb) [file 13287_2018_866_MOESM6_ESM.jpg]
